# Granulocytic sarcoma as an initial manifestation of acute promyelocytic leukemia: A case report with literature review

**DOI:** 10.1097/MD.0000000000041365

**Published:** 2025-02-07

**Authors:** Yuyang Liu, Xiao Huang

**Affiliations:** a The Department of Clinical Laboratory, The Sixth People’s Hospital of Chengdu, Chengdu, China; b The Department of Clinical Laboratory Medicine, West China Hospital, S.U., Chengdu, China.

**Keywords:** acute promyelocytic leukemia, case report, granulocytic sarcoma, humerus

## Abstract

**Rationale:**

Granulocytic sarcoma (GS) is a rare tumor consisting of myeloid blasts with or without maturation and occurs in sites other than the bone marrow. Due to its low prevalence, clinical cases and pathogenesis need to be studied. Therefore, we present a rare case of humerus GS occurring simultaneously with acute promyelocytic leukemia (APL) and studied a retrospective analysis of clinical characteristics and related treatment strategies, hoping that it could help to standardize the early diagnosis and treatment of APL/GS.

**Patient concerns:**

We present a case of humerus GS complicated with APL in a 22-year-old woman who experienced pain in right clavicle and shoulder for 6 months without any cause. While the aggravated pain were persistent for 2 months, causing limited movement of her right upper arm.

**Diagnoses:**

The presence of tumor in her right proximal humerus and end of clavicle was revealed by positron emission tomography–computed tomography. Subsequently, the mass collected during the operation was confirmed to be GS by the pathological immunohistochemical examination. Further progression to APL was based on marrow smears, flow cytometry, fluorescence in situ hybridization, and PML/RARα gene detection.

**Interventions and outcomes:**

The patient underwent the tumorectomy, and then received 28-day induction therapy with all-trans retinoic acid (ATRA) (25 mg/m^2^/d) and arsenic trioxide. The posttreatment bone marrow smear and flow cytometry showed that she was in a complete remission. Consolidation treatment was performed with ATRA 25 mg/m^2^ PO BID for 2 weeks every 4 weeks and arsenic trioxide 0.16 mg/kg IV 5 days a week for 4 weeks every 8 weeks for a total of 6 cycles. Currently, the patient was routinely followed-up at an outpatient clinic, and has been maintained complete remission for 15 months.

**Lessons:**

We present an uncommon case of a humeral APL/GS, and conducted a comprehensive analysis of 28 cases of APL/GS. Despite the rarity of APL/GS, it should be diagnosed at an early stage. Furthermore, ATRA are recommended in the treatment plan of APL/GS.

## 1. Introduction

Granulocytic sarcoma (GS), also known as myeloid sarcoma or chloroma, is an uncommon neoplasm characterized by immature myeloid cells present at an extramedullary site. According to the 5th World Health Organization classification of hematolymphoid tumors, GS can develop primary or occur simultaneously with acute myeloid leukemia (AML), chronic myeloid leukemia, myelodysplastic disorders, and myeloproliferative neoplasms.^[1]^ It may also be an initial manifestation of relapse in AML patients in the remission phase.^[2]^ Studies have indicated that patients diagnosed with myeloid sarcoma comprise 0.8% of all AML diagnoses.^[3]^

Acute promyelocytic leukemia (APL)/GS is a distinct manifestation of APL, comprising promyelocytic cells in extramedullary sites. It accounts for 3% to 5% of APL cases. Similarly, GS as an initial manifestation of APL occurs in < 10% of the GS cases.^[4]^ Owing to the rarity of the disease, contemporary clinical data are mostly limited to small case series. The incidence of APL/GS is unique in that GS occurs across most age groups with a decreased incidence in early childhood and in people over 70 years of age.^[4,5]^ Clinical studies on APL/GS have shown that this disease can occur in various parts of the body, including central nervous system, skin, bone, and tongue.^[6]^ Despite its rarity and uncharacteristic clinical appearance, APL/GS can be easily confused with various malignancies such as small round cell tumors, solid tumors, and thymoma, which may delay diagnosis and treatment.^[7]^ Its presentation requires a quick diagnosis, whereas delayed diagnosis and treatment may worsen a patient’s prognosis. Herein, we report a clinical case of humeral APL/GS and summarized 28 cases of APL/GS management through related researches. This case expands our knowledge of the standardization of APL/GS diagnosis and treatment.

## 2. Case report

**Figure 1.**
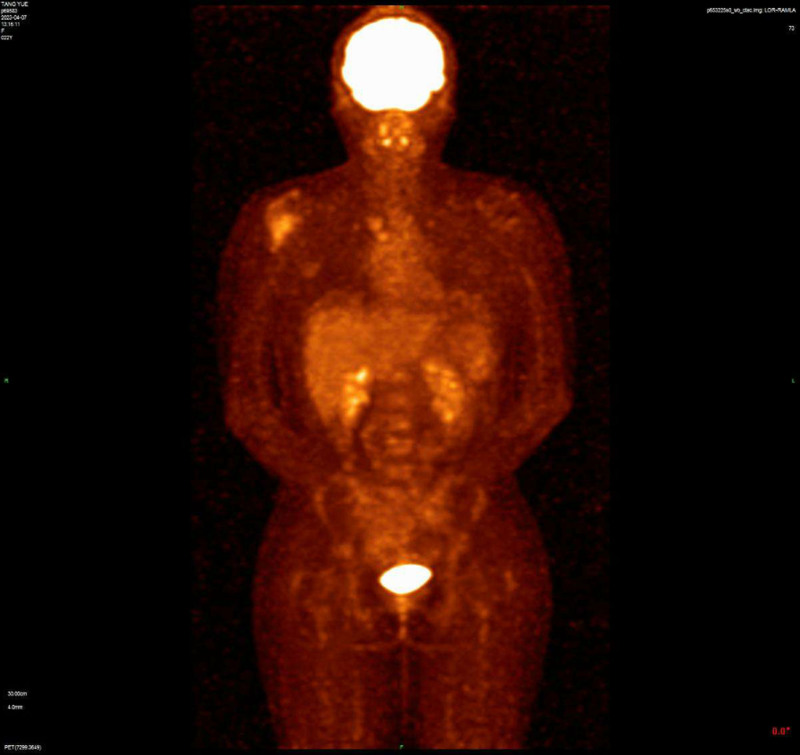
PET-CT revealed the presence of a tumor in the right proximal humerus and end of the clavicle. PET-CT = positron emission tomography–computed tomography.

**Figure 2.**
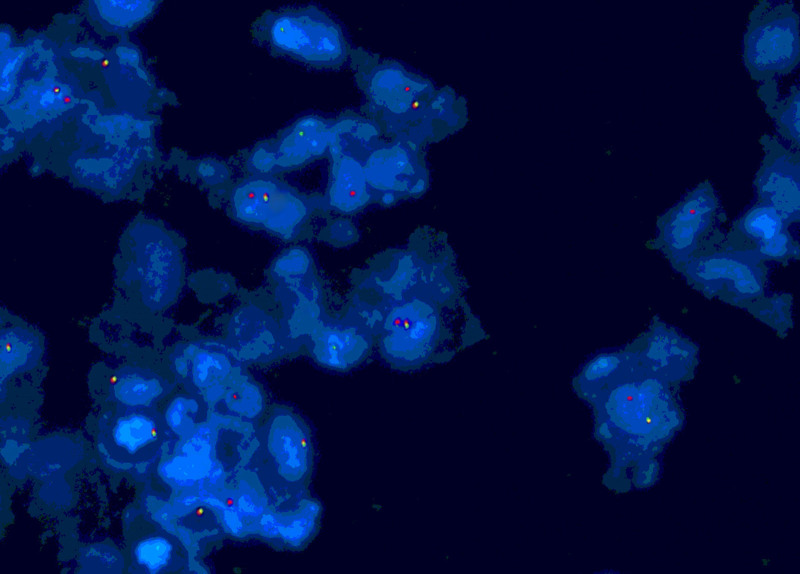
FISH analysis with PML and RARA double fusion probes demonstrating the presence of PML/RARα fusion rearrangement. FISH = fluorescence in situ hybridization.

**Figure 3.**
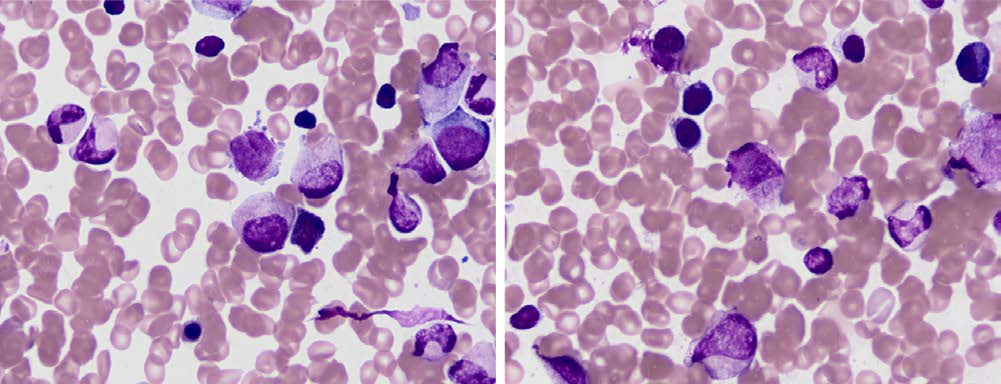
Bone marrow smear showed the proliferation of aberrant promyelocytes (×1000).

**Figure 4.**
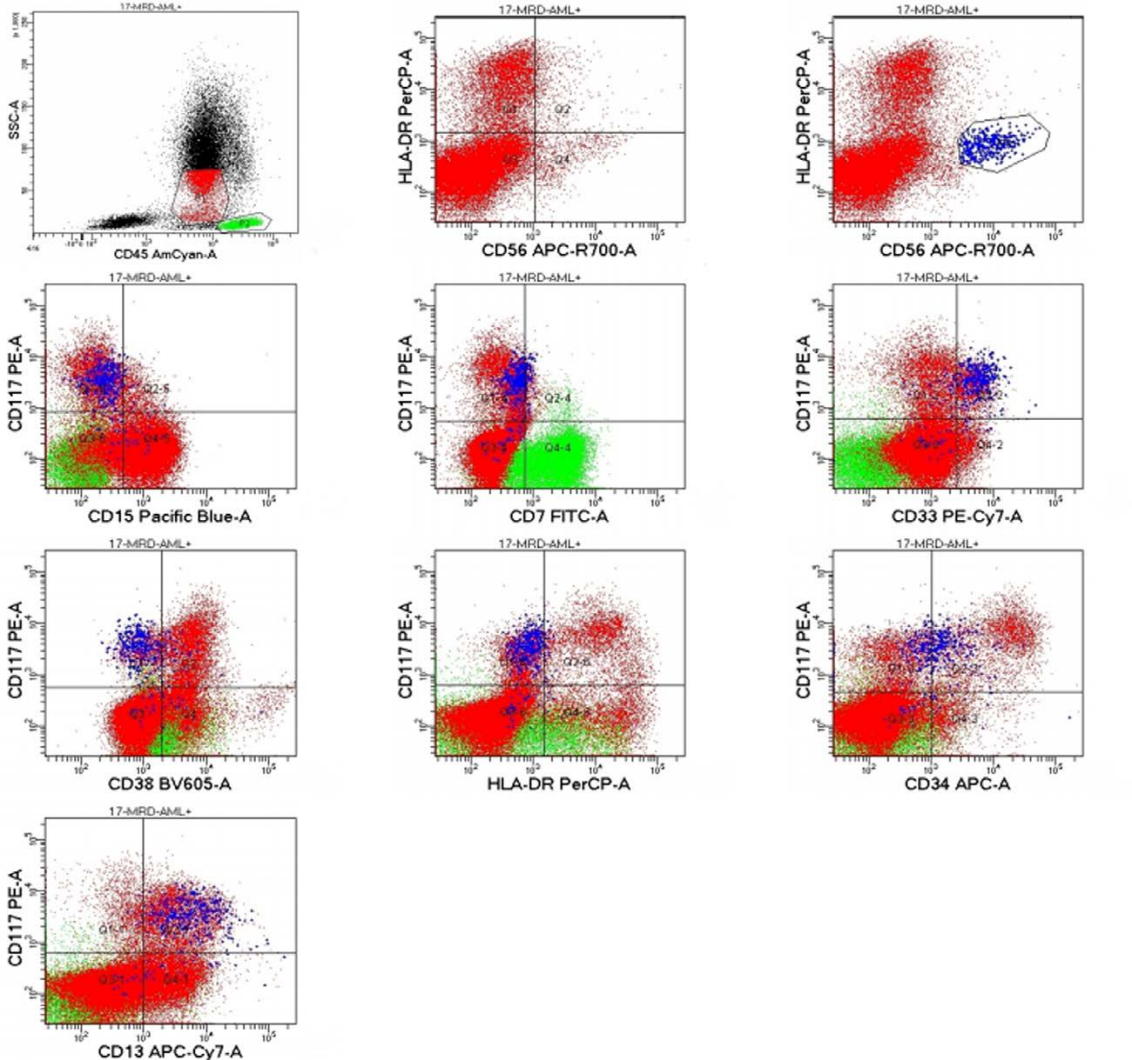
Flow cytometry scatter plots demonstrating the abnormal promyelocytes expressing CD117, CD13, CD33, and CD56, weakly expressing CD34, but not CD15, CD38, or HLA-Dr.

A 22-year-old woman experienced pain in right clavicle and shoulder 6 months ago without any cause, which lasted for a few seconds before disappearing spontaneously. The pain occurred approximately every 3 to 4 days without fever, skin nodules, or restricted mobility, and was not treated. While the aggravated pain were persistent for 2 months, causing limited movement of her right upper arm. The patient has no medical history or family history. She was admitted to the hospital in February 2023, and provided informed consent for both the procedures itself and the publication of the resulting data. A thorax computed tomography scan revealed a pathological mass located in the right proximal humerus and end of the clavicle, which were considered malignant. Furthermore, positron emission tomography/computed tomography confirmed its presence (Fig. 1). She underwent the tumorectomy. The surgical specimen sized 1.2 × 0.8 × 0.4 cm, with segmental lysis and the involvement of the humerus. The pathology was consistent with GS, consisting of immature myeloid cells that staining for CD33 (+), MPO (+), CD99 (+), CD34 (±), TdT (±), CD43 (‐), LCA (‐), CD30 (‐), CD20 (‐), CD3 (‐), CD5 (‐), IgG4 (‐), ALK-V (‐), Oct2 (‐), CD56 (‐), CyclinD1 (‐), CD79a (‐), and ki67 (+, 5%–10%). Concurrent fluorescence in situ hybridization analysis of the tumor was positive for t(15;17) in 14% of the cells, showing a double fusion of PML/RARα along with one normal signal each for PML and RARα, which confirmed the diagnosis (Fig. 2). Pathological promyelocytes (4.0%) were observed in the bone marrow smear (Fig. 3). Myeloperoxidase staining revealed that most blast cells were strongly positive (4/5). Flow cytometry detection of the bone marrow confirmed 0.08% of the abnormal promyelocytes expressing CD117, CD13, CD33, and CD56, weakly expressing CD34, but not CD15, CD38, or HLA-DR (Fig. 4). Polymerase chain reaction for the PML–RARα/ABL gene was positive at 52.2593%. Karyotype analysis also demonstrated the presence of 46,XX,t(15;17)(q24;q21) in 3 cells, with 7 additional cells showing a normal female karyotype (46,XX). While normal results at the time were shown in the coagulation, and complete blood count tests. These results confirmed the coexisting of humeral GS with the early stage of APL.

The patient underwent 28-day induction therapy with all-trans retinoic acid (ATRA) (25 mg/m^2^/d) and arsenic trioxide (ATO) (0.16 mg/kg/d) on March 14th, 2023. During this period, she experienced severe vomiting, fever, granulocyte deficiency, which were resolved with symptomatic treatment. The posttreatment bone marrow smear and flow cytometry showed that she was in a complete remission (CR). The expression of PML–RARα/ABL decreased to 12.0388%. Consolidation treatment was performed with ATRA 25 mg/m^2^ PO BID for 2 weeks every 4 weeks and ATO 0.16 mg/kg IV 5 days a week for 4 weeks every 8 weeks for a total of 6 cycles, which was completed in November 2023. No abnormal metabolic uptake were observed by posttreatment positron emission tomography–computed tomography. Normal results on bone marrow smear, flow cytometry and PML–RARα/ABL gene expression confirmed measurable residual disease negative remission. Currently, the patient was routinely followed-up at an outpatient clinic, and has been maintained CR for 15 months.

## 3. Discussion

As a special manifestation of APL, APL/GS is an extramedullary tumor comprising leukemic cells halted at the promyelocyte stage. Here, we report an unusual site for APL/GS, which has not been previously reported. Despite its inapparent clinical manifestations, histological examination and IH staining are 2 essential modalities for confirming the existence of GS. Furthermore, the detection of the PML/RARα fusion gene and cytogenetic analysis led to an accurate diagnosis of APL/GS. Numerous studies have illustrated that patients who present with APL are at a high risk of developing coagulopathies, which was not observed in our case because of its early detection.

Furthermore, we conducted a comprehensive analysis of 28 cases of APL/GS previously reported in the literature (from 1995 to 2022)^[5,6,8–32]^ at home and abroad. The relevant clinical characteristics and management of APL/GS combined with this case are outlined in Table 1. Of the 29 cases that were identified, the average age at diagnosis was 36 years (range, 1–77 years). The male-to-female ratio was 1.07:1. The central nervous system was the most common site of presentation (11/29). The others were located in the digestive system, ear canal, reproductive system, bone, oral cavity, skin, breast, tongue, breast, and thymus. Our research indicated that primary APL/GS would progress to APL without any leukemia related treatment (3–36 months). Meanwhile, a series of extramedullary infiltration related symptoms could be the main manifestations in GS coexisted with APL. These results supported the studies that GS was a rare but significant clinical feature for leukemia,^[2–4]^ and emphasized the importance of early diagnosis on GS.

**Table 1:** Clinical features, treatment, and outcomes of this case along with 28 cases of APL/GS reported in the literature.

Author	Sex/age	Site of GS	Occurrence of GS in relation to APL	Therapy	Response	Follow-up period
Our case	F/22	Humerus	Concomitant	Surgical + ATO + ATRA	CR	16 mo
Joshua Kra, et al^[6]^	F/23	Mastoid	Secondary	ATRA + CHTH (at first) ATO + RT + Auto-HSCT (at relapse)	2nd CR	16 yr<
Latagliata R, et al^[8]^	F/16	Mastoid	Secondary	CHTH (at first) ATRA + CHTH (at relapse)	2nd CR	NA
Sharon A. Allen, et al^[9]^	F/67	Suprasellar region	Secondary	NA(at first) ATRA + ATO + RT (at relapse)	2nd CR	103 mo<
Latagliata R, et al^[8]^	F/16	Mastoid	Secondary	CHTH (at first) ATRA + CHTH + RT (at relapse)	2nd CR	NA
John R. Krause, et al^[10]^	M/27	Spine	Primary	Surgical + ATRA + ATO + CHTH	CR	16 mo<
Tomoko Yamashita, et al^[5]^	M/50	Spine	Primary	Surgical + ATRA + CHTH	CR	40 mo<
Fiegl M, et al^[11]^	M/55	Vertebra, epidura	Primary	Surgical + ATRA + CHTH + RT	CR	13 mo<
Savranlar A, et al^[12]^	M/18	Epidura	Primary	Surgical + CHTH	PR	NA
Damodar S, et al^[13]^	M/29	Colon	Concomitant	Surgical + ATRA + CHTH	CR	NA
Anna Tosi, et al^[14]^	M/27	Spine	Concomitant	Surgical + ATRA + CHTH + RT	CR	NA
Shu X, et al^[15]^	F/50	Spine	Concomitant	Surgical + ATO + ATRA	CR	10 mo<
Mohamedbhai S, et al^[16]^	M/45	Tongue	Concomitant	Surgical + ATRA + CHTH	CR	NA
Ajarim DS, et al^[17]^	M/21	Thymus	Concomitant	Surgical + CHTH	CR	Died (after 8 mo)
Shanti Gopal, et al^[18]^	M/27	Testicle	Primary	NA	NA	NA
Shintaro F, et al^[19]^	F/36	Cerebellum	Concomitant	Surgical + CHTH	NR	Died (after 4 d)
Wang X, et al^[20]^	F/26	Ovary	Primary	Surgical + ATRA + ATO + CHTH	CR	3 yr<
Benjazia E, et al^[21]^	F/17	Rectum	Concomitant	Surgical + ATRA + ATO + CHTH	CR	4 yr<
Bittencourt H, et al^[22]^	M/53	Spine	Concomitant	Surgical + ATRA + CHTH + RT	CR	Died
Tay Za Kyaw, et al^[23]^	M/26	Spine	Concomitant	Surgical + ATRA + CHTH	CR	1 yr<
Yamashita Y, et al^[24]^	M/1	Mandible	Primary	Surgical + ATRA + CHTH	CR	1 yr<
Piñán MA, et al^[25]^	F/61	Spine	Primary	Surgical + ATRA + RT	CR	8 yr<
Kikuma T, et al^[26]^	M/52	Spine	Concomitant	Surgical + ATRA + CHTH	CR	NA
De Andrade BA, et al^[27]^	F/24	Oral cavity	Concomitant	Surgical + ATRA + CHTH	NA	Died (after 1 mo)
Collinge E, et al^[28]^	F/49	Skin	Concomitant	Surgical + ATO + ATRA	CR	6 mo<
Oravcova I, et al^[29]^	F/34	Breast	Primary	Surgical + ATRA + CHTH	CR	Died (after 5 wk)
Ignacio-Cconchoy FL, et al^[30]^	M/35	Tongue	Concomitant	Surgical + ATRA + CHTH	CR	NA
Wang L, et al^[31]^	F/77	Colon	Concomitant	Surgical + ATRA + CHTH	CR	NA
Harrer DC, et al^[32]^	M/67	Skin	Primary	Surgical + ATRA + CHTH	CR	NA

ATO = arsenic trioxide, ATRA = all-trans retinoic acid, Auto-HSCT = autologous hematopoietic stem cell transplantation, CHTH = chemotherapy, CR = complete remission, d = day, DP = disease progression, F = female, M = male, mo = month, NA = not available, NR = no response, PR = partial response, RT = radiotherapy, wk = week, yr = year.

At present, the treatment options for APL/GS include surgery, ATRA, systemic chemotherapy, radiotherapy, and allogeneic or autologous bone marrow transplantation. It was confirmed that ATRA was the preferred drug for treatment of APL/GS regardless of GS appearance sequence.^[3,4]^ While a few researches showed that the rate of extramedullary relapse has increased with the introduction of ATRA. An ex vivo experiment showed that ATRA could increase expression of cellular adhesion molecules like LFA-1 and VLA-4 on APL cells, which helped APL cells migrating and adhering to extramedullary tissues.^[33]^ In our retrospective study, 25 cases were treated with tumorectomy, ATRA with or without chemotherapy (daunorubicin and cytarabine) or a combination of ATRA and ATO. Most of the patients achieving CR (23/25). While one died from the devastating complications associated with APL.^[22]^ Joshua Kra^[6]^ reported that a case of APL experienced extramedullary recurrence after ATRA combined with chemotherapy treatment. She achieved CR and lived for over 16 year in combination therapy of ATO, radiotherapy and Auto-HSCT. Due to the limited evidence available, we cannot provide sufficient proof supporting a particular therapy that could be considered the gold standard. However, these case reports can provide useful data for therapeutic approaches on APL/GS. The combination therapy of surgery, ATRA with or without chemotherapy or a combination of ATRA and ATO could improve patient survival and prognosis.

In general, we reported a rare case of humerus GS complicated with APL and studied a retrospective analysis of clinical characteristics and treatment strategies related to APL/GS. APL/GS is a rare extramedullary neoplasm with heterogeneous clinical manifestation. This presentation requires a quick diagnosis. Through an in-depth analysis of our cases, along with previous studies concerning APL/GS, we sought to provide further insights into both diagnostic and therapeutic approaches for the management of patients affected by APL/GS. We emphasized that histopathological and molecular diagnostic tools are essential for selecting appropriate therapy. And The combination therapy of surgery, ATRA with or without chemotherapy (daunorubicin and cytarabine) or a combination of ATRA and ATO would be the preferred treatment for various types of APL/GS. Due to the scarcity of relevant reports, more evidence is needed for the standardization of early diagnosis and treatment of APL/GS. Despite its occurrence, physicians should consider GS in patients at risk of hematological malignancies in the presence of pathological masses.

## Author contributions

**Data curation:** Xiao Huang.

**Investigation:** Yuyang Liu.

**Writing – original draft:** Yuyang Liu.

**Writing – review & editing:** Xiao Huang.
